# A Case of Advanced Gastric Cancer With Multiple Liver Metastases in Which Hypoglycemic Symptoms Triggered the Diagnosis

**DOI:** 10.7759/cureus.18407

**Published:** 2021-09-30

**Authors:** Naonori Inoue, Hideaki Kawabata, Masatoshi Miyata

**Affiliations:** 1 Department of Gastroenterology, Kyoto Second Red Cross Hospital, Kyoto, JPN; 2 Department of Gastroenterology, Kyoto Okamoto Memorial Hospital, Kyoto, JPN

**Keywords:** palliative medicine, igf-ii, nicth, hypoglycemia, liver metastases

## Abstract

We experienced a case of gastric cancer with multiple liver metastases characterized by frequent hypoglycemic attacks. Hypoglycemia was observed on admission. We suspected that the cause of this hypoglycemia was non-islet cell tumor hypoglycemia (NICTH). Staining of the tissue with an insulin-like growth factor (IGF)-II antibody revealed that IGF-II was present in the tumor cells. This finding suggested that the tumor was producing IGF-II, which leads to NICTH. After starting parenteral nutrition, the patient emerged from the hypoglycemic coma. He remained out of the coma until he died of liver failure.

## Introduction

Tumor-induced hypoglycemia was first reported in 1929 by Nadler et al. [1]. Since then, it has been accepted that insulin-like active substances are likely to be central to the pathogenesis of hypoglycemia. In 1988, Daughaday et al. reported that insulin-like growth factor (IGF)-II can be produced in leiomyoma with hypoglycemia [2]. The overproduction of IGF-II leads to the condition now known as non-islet cell tumor hypoglycemia (NICTH) due to extrapancreatic tumors and has been linked to various tumors such as sarcoma, gastric cancer, colon cancer, hepatocellular carcinoma, and renal cell carcinoma. IGF-II is a 7.5-kDa peptide that consists of 67 amino acids and is produced mainly in the liver. It is synthesized when pro-IGF-II (156 amino acids) is hydrolyzed by prohormone convertase 4 (PC4) [3]. Pro-IGF-II is synthesized through the processing of the signal peptide of prepro-IGF-II (20.1 kDa, 180 amino acids). Incomplete processing yields incomplete peptides of 10-18 kDa, which are referred to as high-molecular-weight IGF-II. When this high-molecular-weight IGF-II reaches target cells, it binds to insulin receptors and IGF-I receptors and exerts physiological actions such as inhibiting glucose release from the liver and glucose uptake into skeletal muscles. It is also known that glucagon and growth hormone are decreased by increases in IGF-II [4]. Here, we report a case of advanced gastric cancer with multiple liver metastases that produced IGF-II, resulting in frequent hypoglycemic symptoms.

## Case presentation

**Figure 1:**
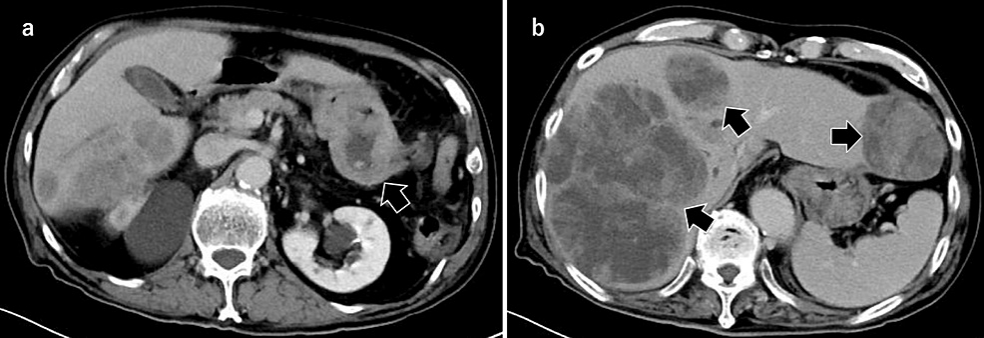
(a) Irregular wall thickening and increased adipose tissue concentration were observed from the gastric body to the angular notch (arrow). (b) Contrast-enhanced computed tomography (CT) revealed multiple tumors (arrows). All tumors appeared as low-density areas on contrast-enhanced CT.

In August 2017, an 85-year-old man was admitted to the Kyoto Okamoto Memorial Hospital due to a disturbance of consciousness (Glasgow coma scale [GCS] E1V2M2). Laboratory data on admission, shown here in Table 1, revealed marked hypoglycemia, iron-deficiency anemia, elevation of hepatobiliary enzymes, and strong inflammatory response. C-peptide, growth hormone (GH), and IGF-I were low. Contrast-enhanced computed tomography (CT) imaging revealed irregular thickening of the stomach wall from the gastric body to the incisura and increased density of the surrounding adipose tissue (Figure 1, Panel a). Multiple large lymph nodes were found near the affected site. In addition, poor contrast enhancement tumors with poor contrast effects were observed in the liver (Figure 1, Panel b), but no abnormalities were observed in other organs including the pancreas. No abnormalities were found in cranial CT.

**Table 1:** Laboratory data on admission AST: Aspartate aminotransferase; ALT: alanine aminotransferase; ALP: alkaline phosphatase; LDH: lactate dehydrogenase; γ-GTP: γ-glutamyltranspeptidase; ChE: cholinesterase; BUN: blood urea nitrogen; Cre: creatinine; CA19-9: carbohydrate antigen 19-9; CEA: carcinoembryonic antigen; IRI: immunoreactive insulin; GH: growth hormone; TSH: thyroid-stimulating hormone; IGF: insulin-like growth factor.

Tests	Patient Value	Normal Value
Total protein	7.1 g/dL	6.5-8.2 g/dL
Albumin	2.9 g/dL	3.7-5.5 g/dL
AST	123 IU/L	10-40 IU/L
ALT	70 IU/L	5-45 IU/L
ALP	908 IU/L	104-338 IU/L
LDH	536 IU/L	120-245 IU/L
Total bilirubin	1.0 mg/dL	0.3-1.2 mg/dL
Gamma-GTP	170 IU/L	0-79 IU/L
ChE	96 IU/L	245-495 IU/L
BUN	7.9 mg/dL	8-20 mg/dL
Cre	0.44 mg/dL	0.65-1.09 mg/dL
Sodium	143 mEq/L	135-145 mg/dL
Potassium	3.5 mEq/L	3.5-5 mg/dL
Plasma glucose	26 mg/dL	70-109 mg/dL
Hemoglobin A1c	5.3%	4.6%-6.2%
White blood cells	12 x 10^9^/L	3.5-9.7 x 10^9^/L
Red blood cells	313 x 10^10^/L	438-577 x 10^10^/L
Hemoglobin	7.7 g/dL	13.6-18.3 g/dL
Hematocrit	26.0%	40.4-51.9%
Platelets	41.8 x 10^10^/L	14-38 x 10^10^/L
CA19-9	36.6 U/mL	<37.0 U/mL
CEA	4.9 ng/mL	<5.0 ng/mL
IRI	2.8 pmol/L	2.2-12.4 pmol/L
C-peptide	0.1 ng/mL	0.8-2.5 ng/mL
Cortisol	265 nmol/L	120-582 nmol/L
GH	0.06 ng/mL	<2.47 ng/mL
TSH	0.63 mIU/mL	0.34-4.22 mIU/mL
Free T_3_	1.76 pg/mL	2.24-3.94 pg/mL
Free T_4_	1.29 ng/dL	0.77-1.59 ng/dL
IGF-1	21 ng/mL	48-177 ng/mL

**Figure 2:**
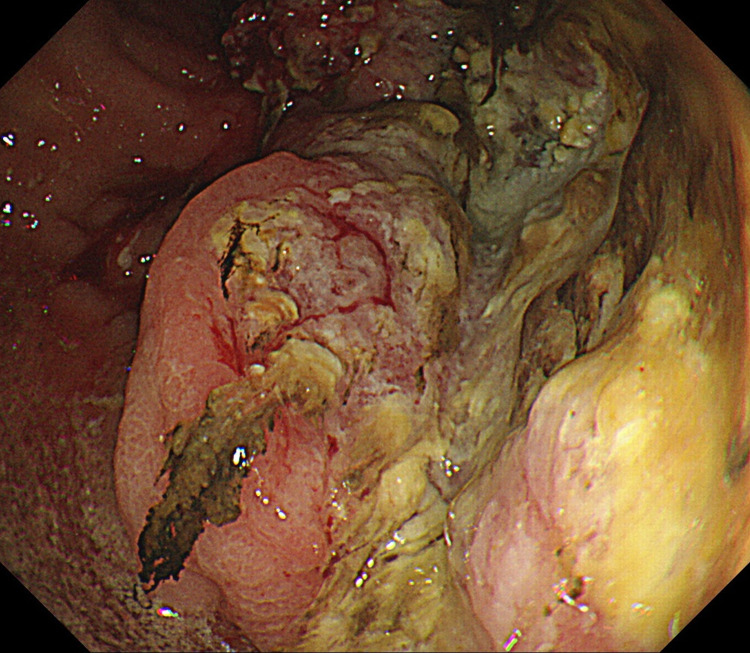
Esophagogastroduodenoscopy revealed a hemorrhagic type 2 advanced cancer in the posterior wall of the gastric body

**Figure 3:**
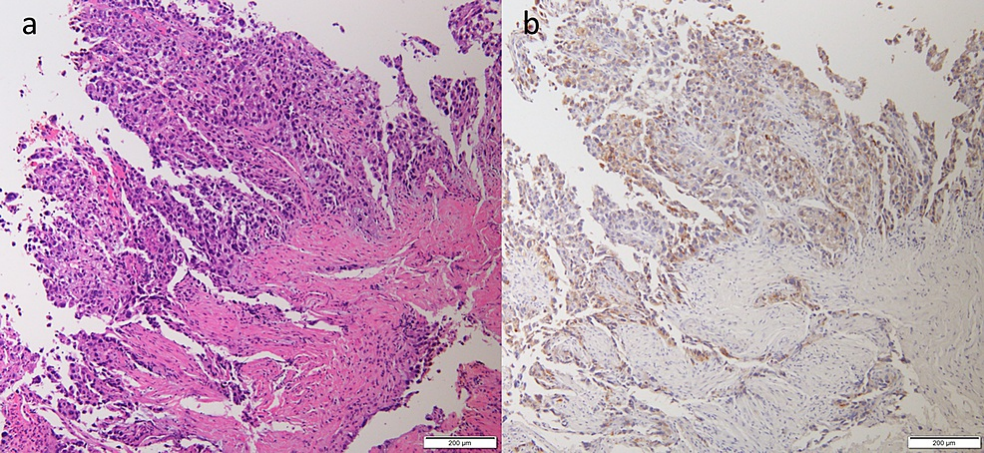
(a) Biopsied section of the gastric tumor stained with hematoxylin–eosin (HE), which was pathologically diagnosed as poorly differentiated adenocarcinoma. (b) Biopsied section of the gastric tumor stained with insulin-like growth factor (IGF)-II, which revealed that IGF-II was present at the location of the tumor cells.

Esophagogastroduodenoscopy revealed advanced cancer that bled easily in the posterior wall of the gastric body (Figure 2). A biopsy from the same site was found to be poorly differentiated adenocarcinoma (Figure 3, Panel a). Based on these findings, the patient was diagnosed with advanced gastric cancer with multiple liver metastases. In addition, immunostaining with anti-IGF-II antibody revealed that IGF-II was present at the location of the tumor cells (Figure 3, Panel b). Due to his age and poor functional status, after discussion with his family, it was determined that he was not indicated for active treatment.

Since the patient’s anemia was considered to be caused by bleeding from the gastric tumor, palliative radiation therapy of 30 Gy in 10 fractions (3 Gy/daily) was performed to achieve hemostasis. After irradiation, his anemia did not progress.

When we treated his hypoglycemia by administering glucose, his disturbance of consciousness gradually improved (GCS E4V5M6). For some time after admission, he was able to manage his hypoglycemic attacks, which tended to occur early in the morning, by self-administering oral glucose. A week after admission, however, food intake became difficult for him, and his hypoglycemic attacks became more frequent such that he required 180-240 g/day of glucose. At that point, it became difficult to maintain a normal blood glucose level even with peripheral continuous parenteral nutrition containing 200 g/day of glucose, and he entered a prolonged state of disturbance of consciousness (GCS E1V2M2). At this time, his Karnofsky Performance Status (KPS) was 10-20.

After consulting with his family, we started central parenteral nutrition containing 250 g/day of glucose despite the patient being in the terminal stage by the 18th day after admission. This palliative treatment stabilized his blood glucose level at around 80 mg/dL and eliminated his hypoglycemic symptoms. His KPS score temporarily improved to 30. He was able to enjoy talking with his family and eating small amounts of food until he died of liver failure on the 26th day after admission.

## Discussion

Hypoglycemic symptoms that emerge in connection with tumors are often attributed to insulinoma, but in cases with no increase in endogenous insulin, NICTH may be the cause [5]. NICTH is induced when tumors produce excessive quantities of the hormone IGF-II. Leiomyosarcoma with hypoglycemia, for example, exhibits high concentrations of IGF-II mRNA and higher than normal levels of IGF-II as Daughaday et al. have reported [2].

NICTH is typically associated not only with hypoglycemia but also with low levels of insulin, proinsulin, C-peptide, beta-hydroxybutyrate, and GH. In addition, IGF binding protein 3 and acid-labile subunit are also decreased along with growth hormone [6]. The IGF-II value is not always high, but the IGF-I value is often low, resulting in a high IGF-II:IGF-I ratio [7]. At most medical institutions, however, it is difficult to measure serum IGF-II as sodium dodecyl sulfate-polyacrylamide gel electrophoresis (SDS-PAGE) with western immunoblotting is required to confirm the presence of high-molecular-weight IGF-II. In clinical settings that lack this technology, it is necessary to diagnose NICTH indirectly from clinical data. In practice, NICTH should be suspected whenever there is a large tumor burden, hypoglycemia, low blood insulin, and low IGF-I levels.

When hypoglycemia is associated with liver tumors, it may be difficult to determine if hypoglycemia may be due to glycogen depletion in the liver, which occurs as a result of liver failure. One measurement that can help distinguish the etiology of hypoglycemia in such cases is the change in blood glucose level due to glucagon load [8,9]. In the present case, there was no opportunity to perform the glucagon load test, but the tumor occupancy rate in the liver was high, reaching approximately 60% on CT, suggesting that liver glycogen may have been reduced. Staining with an antibody to IGF-II returned positive findings in the pathological tissue from the gastric biopsy. Given that blood insulin, C-peptide, IGF-I, and GH were all low and that a relatively large tumor external to the pancreas was visible in imaging, we diagnosed the patient with NICTH. But because this antibody is not specific to high-molecular-weight IGF-II, this finding does not definitively indicate the presence of high-molecular-weight IGF-II.

The fundamental treatment for NICTH is complete excision of the tumor, but in cases where this is not possible, glucose is administered orally and intravenously as needed. The administration of glucagon and recombinant human GH can also improve hypoglycemia [10-12]. Administration of steroids has been shown to be effective in some cases [13,14], but in other cases with liver metastasis, no effect is observed [15]. In this case, hypoglycemic symptoms disappeared upon starting intravenous hyperalimentation, but in cases with a long-term treatment course, the administration of steroids or glucagon may be a viable treatment. There is no evidence that high-calorie supplemental infusions extend survival in end-stage cancer patients without gastrointestinal obstructions, and such infusions are not recommended as they are likely to cause metabolic complications [16].

In this case, however, the patient’s quality of life was improved by alleviating his hypoglycemic symptoms with a high-calorie infusion. This palliative treatment enabled him to spend a brief period of time calmly interacting with his family, which is a highly desirable outcome in end-stage cancer. Studies have been conducted on whether fluid infusions for end-stage cancer patients improve the quality of life. High-calorie infusion for patients with KPS of 40 or higher showed an improvement in the quality of life [17]. In addition, it has been reported that high-calorie infusion helped in achieving patient/family satisfaction and maintenance of performance status [18] in multiple case reports. However, it has been reported that patients with the worse general conditions were able to maintain overall premortal comfort at 84% without the use of fluids at all [19]. According to the guidelines agreed by experts, the indications for high-calorie infusion include a life prognosis of 40-60 days or more, patients with a life prognosis of t months or more, KPS of 40 or more, and performance status of 0-2 [20]. In this case, KPS was 10-20, and high-calorie infusion was not recommended. However, high-calorie infusion improved KPS to 30. Depending on the situation, high-calorie infusion to terminal cancer patients, as in this case, maybe considered.

## Conclusions

In conclusion, we experienced a case of NICTH due to IGF-II production in multiple liver metastases of gastric cancer with frequent hypoglycemic attacks. We found that a high-calorie infusion was useful for alleviating the patient’s symptoms and improving his quality of life.

## References

[1] Nadler WH, Wolfer JA (1929). Hepatogenic hypoglycemia associated with primary liver cell carcinoma. Arch Intern Med.

[2] Daughaday WH, Emanuele MA, Brooks MH, Barbato AL, Kapadia M, Rotwein P (1988). Synthesis and secretion of insulin-like growth factor II by a leiomyosarcoma with associated hypoglycemia. N Engl J Med.

[3] Livingstone C (2013). IGF2 and cancer. Endocr Relat Cancer.

[4] LeRoith D, Roberts CT Jr (2003). The insulin-like growth factor system and cancer. Cancer Lett.

[5] Takayama-Hasumi S, Eguchi Y, Sato A, Morita C, Hirata Y (1990). Insulin autoimmune syndrome is the third leading cause of spontaneous hypoglycemic attacks in Japan. Diabetes Res Clin Pract.

[6] Fukuda I, Hizuka N, Takano K, Asakawa-Yasumoto K, Shizume K, Demura H (1993). Characterization of insulin-like growth factor II (IGF-II) and IGF binding proteins in patients with non-islet-cell tumor hypoglycemia. Endocr J.

[7] Fukuda I, Hizuka N, Ishikawa Y (2006). Clinical features of insulin-like growth factor-II producing non-islet-cell tumor hypoglycemia. Growth Horm IGF Res.

[8] Rastogi MV, Desai N, Quintos JB (2008). Non-islet-cell tumor hypoglycemia and lactic acidosis in a child with congenital HIV and Burkitt's lymphoma. J Pediatr Endocrinol Metab.

[9] Kishi K, Homma S, Tanimura S, Matsushita H, Nakata K (2001). Hypoglycemia induced by secretion of high molecular weight insulin-like growth factor-II from a malignant solitary fibrous tumor of the pleura. Intern Med.

[10] Föger B, Zapf J, Lechleitner M, Konwalinka G, Patsch JR (1994). Prevention with glucocorticoids of extrapancreatic tumour-hypoglycaemia as a result of increased 'big' insulin-like growth factor II. J Intern Med.

[11] Powter L, Phillips S, Husbands E (2013). A case report of non-islet cell tumour hypoglycaemia associated with ovarian germ-cell tumour. Palliat Med.

[12] Mukherjee S, Diver M, Weston PJ (2005). Non islet cell tumor hypoglycaemia in a metastatic Leydig cell tumor. Acta Oncol.

[13] Tsuro K, Kojima H, Okamoto S (2006). Glucocorticoid therapy ameliorated hypoglycemia in insulin-like growth factor-II-producing solitary fibrous tumor. Intern Med.

[14] Baxter RC, Holman SR, Corbould A, Stranks S, Ho PJ, Braund W (1995). Regulation of the insulin-like growth factors and their binding proteins by glucocorticoid and growth hormone in nonislet cell tumor hypoglycemia. J Clin Endocrinol Metab.

[15] Kato A, Bando E, Shinozaki S (2004). Severe hypoglycemia and hypokalemia in association with liver metastases of gastric cancer. Intern Med.

[16] Morita T, Hyodo I, Yoshimi T (2005). Association between hydration volume and symptoms in terminally ill cancer patients with abdominal malignancies. Ann Oncol.

[17] King LA, Carson LF, Konstantinides N (1993). Outcome assessment of home parenteral nutrition in patients with gynecologic malignancies: what have we learned in a decade of experience?. Gynecol Oncol.

[18] Chapman C, Bosscher J, Remmenga S, Park R, Barnhill D (1991). A technique for managing terminally ill ovarian carcinoma patients. Gynecol Oncol.

[19] McCann RM, Hall WJ, Groth-Juncker A (1994). Comfort care for terminally ill patients. The appropriate use of nutrition and hydration. JAMA.

[20] Bachmann P, Marti-Massoud C, Blanc-Vincent MP (2003). Summary version of the Standards, Options and Recommendations for palliative or terminal nutrition in adults with progressive cancer (2001). Br J Cancer.

